# Diaquadi-μ-formato-bis­{μ-2,2′-[propane-1,3-diylbis(nitrilo­methanylyl­idene)]diphenolato}cadmium(II)dinickel(II) dihydrate

**DOI:** 10.1107/S1600536812029583

**Published:** 2012-07-28

**Authors:** Jian-Feng Zhang, Bo Wan, Wen Liu, Qian Shi

**Affiliations:** a College of Chemistry and Materials Engineering, Wenzhou University, Wenzhou 325035, People’s Republic of China

## Abstract

In the centrosymmetric title compound, [CdNi_2_(C_17_H_16_N_2_O_2_)_2_(HCOO)_2_(H_2_O)_2_]·2H_2_O, The Ni^II^ cation is chelated by a 2,2′-[propane-1,3-diylbis(nitrilo­methanylyl­idene)]diphen­olate (salpn) anion, and further coordinated by a formate anion and a water mol­ecule in a distorted NiN_2_O_4_ octa­hedral geometry. The Cd^II^ cation, located on an inversion center, is coordinated by four deprotonated hy­droxy groups from two salpn anions and two carboxyl­ate O atoms from formate anions in a distorted octa­hedral geometry. Both formate and salpn anions bridge the Cd and Ni cations, forming a trinuclear complex. Within the salpn anion, the benzene rings are twisted to each other at a dihedral angle of 61.46 (18)°. Inter­molecular O—H⋯O hydrogen bonding is present in the crystal structure. The lattice water mol­ecule is disorder over two positions with an occupancy ratio of 0.75:0.25.

## Related literature

For background and applications of metal complexes with Schiff base ligands, see: Niederhoffer *et al.* (1984 ▶); Tisato *et al.* (1994 ▶); Yamada (1999 ▶). For the decomposition reaction of solvent DMF, see: Wang *et al.* (2004 ▶); Zhang *et al.* (2007 ▶).
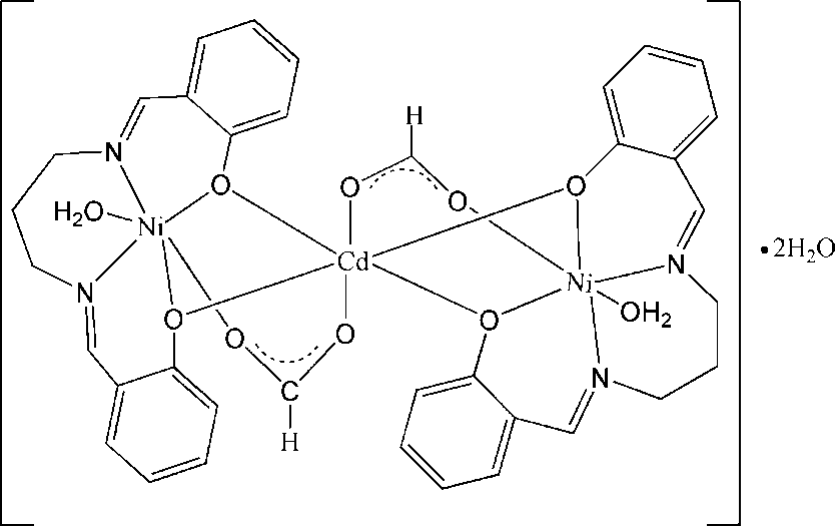


## Experimental

### 

#### Crystal data

[CdNi_2_(C_17_H_16_N_2_O_2_)_2_(HCO_2_)_2_(H_2_O)_2_]·2H_2_O
*M* *_r_* = 952.56
Triclinic, 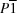

*a* = 9.6769 (9) Å
*b* = 10.6596 (10) Å
*c* = 10.7996 (10) Å
α = 72.851 (1)°
β = 63.551 (1)°
γ = 81.478 (1)°
*V* = 952.87 (15) Å^3^
*Z* = 1
Mo *K*α radiation
μ = 1.60 mm^−1^
*T* = 298 K
0.26 × 0.20 × 0.19 mm

#### Data collection

Bruker SMART 1000 diffractometer
Absorption correction: multi-scan (*SADABS*; Bruker, 2002 ▶) *T*_min_ = 0.681, *T*_max_ = 0.751
4842 measured reflections
3423 independent reflections
2910 reflections with *I* > 2σ(*I*)
*R*_int_ = 0.013

#### Refinement

*R*[*F* ^2^ > 2σ(*F* ^2^)] = 0.026
*wR*(*F* ^2^) = 0.074
*S* = 1.06
3423 reflections
259 parameters
H-atom parameters constrained
Δρ_max_ = 0.46 e Å^−3^
Δρ_min_ = −0.38 e Å^−3^

### 

Data collection: *SMART* (Bruker, 2002 ▶); cell refinement: *SAINT* (Bruker, 2002 ▶); data reduction: *SAINT*; program(s) used to solve structure: *SHELXTL* (Sheldrick, 2008 ▶); program(s) used to refine structure: *SHELXTL*; molecular graphics: *SHELXTL*; software used to prepare material for publication: *SHELXTL*.

## Supplementary Material

Crystal structure: contains datablock(s) I, global. DOI: 10.1107/S1600536812029583/xu5190sup1.cif

Structure factors: contains datablock(s) I. DOI: 10.1107/S1600536812029583/xu5190Isup2.hkl

Additional supplementary materials:  crystallographic information; 3D view; checkCIF report

## Figures and Tables

**Table 1:** Selected bond lengths (Å)

Cd1—O1	2.2809 (18)
Cd1—O2	2.2799 (18)
Cd1—O4	2.300 (2)
Ni1—O1	2.0098 (19)
Ni1—O2	2.0313 (19)
Ni1—O3	2.080 (2)
Ni1—O5	2.205 (2)
Ni1—N1	2.035 (2)
Ni1—N2	2.026 (2)

**Table 2:** Hydrogen-bond geometry (Å, °)

*D*—H⋯*A*	*D*—H	H⋯*A*	*D*⋯*A*	*D*—H⋯*A*
O5—H5*A*⋯O6′^i^	0.85	2.04	2.662 (12)	130
O5—H5*B*⋯O6^i^	0.85	2.29	2.812 (4)	120
O6—H6*B*⋯O4^ii^	0.85	1.98	2.737 (4)	147
O6′—H6′*B*⋯O4^ii^	0.85	2.19	2.769 (12)	125

Symmetry codes: (i) 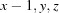; (ii) 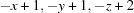.

**Fig. 1.**
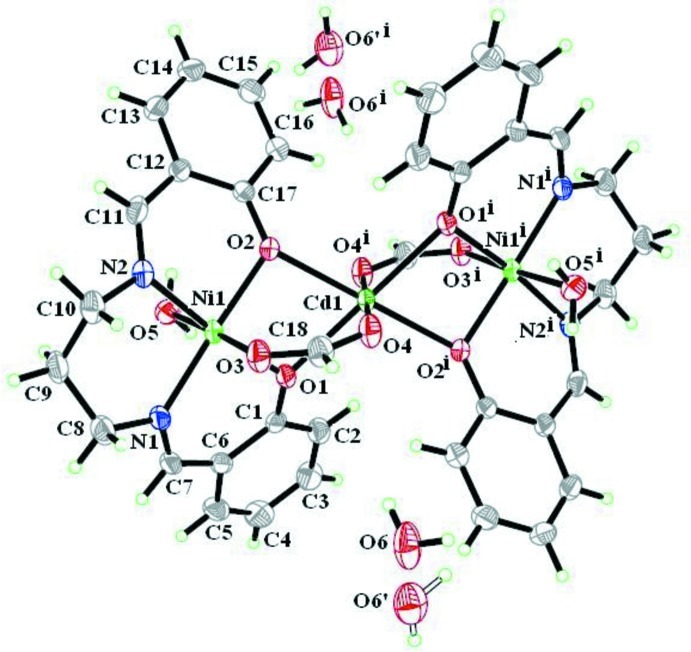
The molecular structure of the title compound showing displacement ellipsoids at 30% probability level [symmetry code: (i) -x, 1-y, 2-z].

## supplementary crystallographic information

### Comment

The molecular design and synthesis of Ni(II) complexes with the salen type Schiff-base ligands have attracted much attention in the past few years (Niederhoffer *et al.*, 1984; Tisato *et al.*, 1994; Yamada, 1999). Hererin we reported the structure of the title complex containing the Schiff base compound, N,N'-bis(salicylidene)-1,3-propanediaminato (salpn). In the compound, the formate anion may be generated from the decomposition of DMF solvents in solvothermal conditions, it has been reported by Wang *et al.* (2004) and by Zhang *et al.* (2007) previously.

In the title compound, the Cd(II) ion is situated on an inversion centre and two terminal Ni(II) ions are located on the symmetrical sides, forming a linear Ni—Cd—Ni trinuclear complex (Fig. 1). The Cd(II) ion has a distorted octahedral coordination environment, formed by four O atoms from two salpn ligands in the equatorial plane and two O atoms from two formate ligands at the axial positions. The coordination bond lengths and angles around the Cd(II) ion range between 2.2799 (18)–2.300 (2) Å, and 73.23 (7)–106.77 (7)°, respectively. The terminal Ni(II) ions have slightly distorted octahedral coordination environments formed by two O atoms and two N atoms from salpn ligands in the equatorial plane and two O atoms from formate ligand and auqa at the axial positions. In the Ni coordination sphere bond lengths and angles range between 2.0098 (2)–2.205 (2) Å, and 84.62 (8) - 177.57 (8)°, respectively. Each pair of metal ions is triply bridged *via* O atoms from salpn ligands and formate ligands. The crystal structure is stabilized by weak O—H···O hydrogen bonds.

### Experimental

A mixture of Cd(NO_3_)_2_^.^4H_2_O (0.125 mmol, 0.0418 g), Ni(NO_3_)_2_^.^6H_2_O (0.125 mmol, 0.0347 g), 1,3-diaminopropane (0.125 mmol, 0.0102 g), salicyladehyde (0.300 mmol, 0.0366 g), DMF (5 ml), CH_3_OH (5 ml) and ditilled water in a 30 ml Telfon-lined reactor was heated at 373 K for two days. After cooling to room temperature, green crystals are obtained for X-ray analysis.

### Refinement

All H atoms were positioned geometrically with C—H = 0.93 (aromatic), 0.97 Å (methylene) and O—H = 0.85 Å, and allowed to ride in their parent atoms with *U*_iso_(H) = 1.2*U*_eq_(C) or 1.5*U*_eq_(O). The lattice water molecule is disorder over two sites, occupancies were fixed as 0.75 and 0.25 for two components.

### Crystal data

**Table:**

[CdNi_2_(C_17_H_16_N_2_O_2_)_2_(HCO_2_)_2_(H_2_O)_2_]·2H_2_O	*Z* = 1
*M**_r_* = 952.56	*F*(000) = 486
Triclinic, *P*1	*D*_x_ = 1.660 Mg m^−^^3^
Hall symbol: -P 1	Mo *K*α radiation, λ = 0.71073 Å
*a* = 9.6769 (9) Å	Cell parameters from 2961 reflections
*b* = 10.6596 (10) Å	θ = 2.4–27.5°
*c* = 10.7996 (10) Å	µ = 1.60 mm^−^^1^
α = 72.851 (1)°	*T* = 298 K
β = 63.551 (1)°	Block, green
γ = 81.478 (1)°	0.26 × 0.20 × 0.19 mm
*V* = 952.87 (15) Å^3^	

### Data collection

**Table:**

Bruker SMART 1000 diffractometer	3423 independent reflections
Radiation source: fine-focus sealed tube	2910 reflections with *I* > 2σ(*I*)
Graphite monochromator	*R*_int_ = 0.013
φ and ω scan	θ_max_ = 25.3°, θ_min_ = 2.0°
Absorption correction: multi-scan (*SADABS*; Bruker, 2002)	*h* = −11→11
*T*_min_ = 0.681, *T*_max_ = 0.751	*k* = −12→12
4842 measured reflections	*l* = −12→11

### Refinement

**Table:**

Refinement on *F*^2^	Primary atom site location: structure-invariant direct methods
Least-squares matrix: full	Secondary atom site location: difference Fourier map
*R*[*F*^2^ > 2σ(*F*^2^)] = 0.026	Hydrogen site location: inferred from neighbouring sites
*wR*(*F*^2^) = 0.074	H-atom parameters constrained
*S* = 1.06	*w* = 1/[σ^2^(*F*_o_^2^) + (0.035*P*)^2^ + 0.566*P*] where *P* = (*F*_o_^2^ + 2*F*_c_^2^)/3
3423 reflections	(Δ/σ)_max_ < 0.001
259 parameters	Δρ_max_ = 0.46 e Å^−^^3^
0 restraints	Δρ_min_ = −0.38 e Å^−^^3^

### Special details

**Table:**

Geometry. All e.s.d.'s (except the e.s.d. in the dihedral angle between two l.s. planes) are estimated using the full covariance matrix. The cell e.s.d.'s are taken into account individually in the estimation of e.s.d.'s in distances, angles and torsion angles; correlations between e.s.d.'s in cell parameters are only used when they are defined by crystal symmetry. An approximate (isotropic) treatment of cell e.s.d.'s is used for estimating e.s.d.'s involving l.s. planes.
Refinement. Refinement of *F*^2^ against ALL reflections. The weighted *R*-factor *wR* and goodness of fit *S* are based on *F*^2^, conventional *R*-factors *R* are based on *F*, with *F* set to zero for negative *F*^2^. The threshold expression of *F*^2^ > σ(*F*^2^) is used only for calculating *R*-factors(gt) *etc*. and is not relevant to the choice of reflections for refinement. *R*-factors based on *F*^2^ are statistically about twice as large as those based on *F*, and *R*- factors based on ALL data will be even larger.

### Fractional atomic coordinates and isotropic or equivalent isotropic displacement parameters (Å^2^)

**Table:**

	*x*	*y*	*z*	*U*_iso_*/*U*_eq_	Occ. (<1)
Cd1	0.0000	0.5000	1.0000	0.03673 (10)	
Ni1	0.14492 (4)	0.34996 (3)	0.74567 (3)	0.03560 (11)	
N1	0.3127 (3)	0.3988 (3)	0.5419 (3)	0.0436 (6)	
N2	0.1331 (3)	0.1589 (2)	0.7553 (3)	0.0422 (6)	
C18	0.3150 (4)	0.3266 (3)	0.9271 (3)	0.0505 (8)	
H18	0.4012	0.2946	0.9443	0.061*	
C1	0.1565 (3)	0.6410 (3)	0.6481 (3)	0.0400 (6)	
C2	0.0920 (4)	0.7624 (3)	0.6718 (3)	0.0513 (8)	
H2A	0.0320	0.7688	0.7651	0.062*	
C3	0.1151 (4)	0.8742 (3)	0.5589 (4)	0.0612 (9)	
H3A	0.0695	0.9538	0.5774	0.073*	
C4	0.2056 (5)	0.8674 (4)	0.4197 (4)	0.0648 (10)	
H4A	0.2214	0.9421	0.3441	0.078*	
C5	0.2714 (4)	0.7498 (4)	0.3942 (3)	0.0564 (9)	
H5	0.3332	0.7463	0.3002	0.068*	
C6	0.2496 (3)	0.6343 (3)	0.5041 (3)	0.0414 (7)	
C7	0.3309 (3)	0.5174 (3)	0.4631 (3)	0.0464 (7)	
H7A	0.4049	0.5294	0.3690	0.056*	
C8	0.4152 (4)	0.2974 (4)	0.4767 (4)	0.0570 (9)	
H8A	0.4948	0.2743	0.5114	0.068*	
H8B	0.4655	0.3332	0.3738	0.068*	
C9	0.3312 (4)	0.1736 (3)	0.5090 (3)	0.0560 (8)	
H9A	0.2448	0.1979	0.4835	0.067*	
H9B	0.4010	0.1178	0.4496	0.067*	
C10	0.2717 (4)	0.0959 (3)	0.6650 (3)	0.0538 (8)	
H10A	0.2475	0.0077	0.6736	0.065*	
H10B	0.3515	0.0891	0.6979	0.065*	
C11	0.0120 (4)	0.0914 (3)	0.8338 (3)	0.0456 (7)	
H11A	0.0181	0.0056	0.8270	0.055*	
C12	−0.1348 (3)	0.1337 (3)	0.9328 (3)	0.0413 (6)	
C13	−0.2637 (4)	0.0565 (3)	0.9825 (3)	0.0548 (8)	
H13A	−0.2512	−0.0186	0.9512	0.066*	
C14	−0.4076 (4)	0.0884 (4)	1.0757 (4)	0.0604 (9)	
H14A	−0.4920	0.0372	1.1051	0.072*	
C15	−0.4247 (4)	0.1982 (4)	1.1251 (3)	0.0561 (8)	
H15A	−0.5218	0.2211	1.1879	0.067*	
C16	−0.3002 (4)	0.2741 (3)	1.0828 (3)	0.0481 (7)	
H16A	−0.3143	0.3455	1.1204	0.058*	
C17	−0.1523 (3)	0.2462 (3)	0.9842 (3)	0.0381 (6)	
O1	0.1331 (2)	0.53609 (19)	0.75777 (19)	0.0421 (5)	
O2	−0.0344 (2)	0.32089 (19)	0.9431 (2)	0.0417 (5)	
O3	0.3110 (2)	0.2997 (2)	0.8257 (2)	0.0511 (5)	
O4	0.2196 (3)	0.3909 (2)	1.0107 (2)	0.0526 (5)	
O5	−0.0232 (2)	0.4059 (2)	0.6516 (2)	0.0500 (5)	
H5A	−0.0308	0.4888	0.6383	0.075*	
H5B	−0.1151	0.3808	0.7073	0.075*	
O6	0.7024 (5)	0.5266 (5)	0.8119 (5)	0.0835 (12)	0.75
H6A	0.6220	0.4853	0.8354	0.125*	0.75
H6B	0.7155	0.5206	0.8862	0.125*	0.75
O6'	0.7860 (15)	0.6031 (14)	0.7325 (14)	0.080 (3)	0.25
H6'A	0.8187	0.6774	0.6749	0.120*	0.25
H6'B	0.7223	0.6136	0.8141	0.120*	0.25

### Atomic displacement parameters (Å^2^)

**Table:**

	*U*^11^	*U*^22^	*U*^33^	*U*^12^	*U*^13^	*U*^23^
Cd1	0.04646 (18)	0.03992 (17)	0.02905 (16)	−0.00391 (12)	−0.01648 (13)	−0.01410 (12)
Ni1	0.0396 (2)	0.0408 (2)	0.0300 (2)	−0.00399 (15)	−0.01343 (16)	−0.01492 (15)
N1	0.0385 (13)	0.0612 (17)	0.0360 (13)	−0.0067 (11)	−0.0126 (11)	−0.0221 (12)
N2	0.0485 (14)	0.0436 (14)	0.0400 (13)	0.0036 (11)	−0.0197 (12)	−0.0190 (11)
C18	0.0474 (17)	0.063 (2)	0.0515 (19)	0.0053 (15)	−0.0289 (15)	−0.0198 (16)
C1	0.0450 (16)	0.0472 (17)	0.0335 (14)	−0.0146 (13)	−0.0195 (13)	−0.0074 (12)
C2	0.067 (2)	0.0481 (18)	0.0425 (17)	−0.0047 (15)	−0.0257 (16)	−0.0108 (14)
C3	0.081 (2)	0.050 (2)	0.059 (2)	−0.0018 (17)	−0.039 (2)	−0.0084 (16)
C4	0.084 (3)	0.057 (2)	0.050 (2)	−0.0139 (19)	−0.0354 (19)	0.0084 (17)
C5	0.056 (2)	0.076 (2)	0.0336 (16)	−0.0190 (17)	−0.0180 (15)	−0.0025 (16)
C6	0.0412 (15)	0.0542 (18)	0.0317 (14)	−0.0138 (13)	−0.0167 (12)	−0.0069 (13)
C7	0.0400 (16)	0.070 (2)	0.0305 (14)	−0.0145 (15)	−0.0100 (13)	−0.0168 (15)
C8	0.0408 (17)	0.080 (2)	0.053 (2)	−0.0002 (16)	−0.0107 (15)	−0.0364 (18)
C9	0.057 (2)	0.067 (2)	0.0493 (19)	0.0056 (16)	−0.0175 (16)	−0.0351 (17)
C10	0.0561 (19)	0.056 (2)	0.057 (2)	0.0106 (15)	−0.0255 (16)	−0.0295 (16)
C11	0.066 (2)	0.0333 (15)	0.0457 (17)	−0.0001 (14)	−0.0289 (16)	−0.0145 (13)
C12	0.0546 (17)	0.0387 (15)	0.0327 (14)	−0.0104 (13)	−0.0201 (13)	−0.0052 (12)
C13	0.074 (2)	0.0514 (19)	0.0453 (18)	−0.0232 (16)	−0.0268 (17)	−0.0083 (15)
C14	0.060 (2)	0.073 (2)	0.0476 (19)	−0.0314 (18)	−0.0198 (17)	−0.0063 (17)
C15	0.0500 (18)	0.074 (2)	0.0412 (17)	−0.0136 (16)	−0.0160 (15)	−0.0095 (16)
C16	0.0530 (18)	0.0515 (18)	0.0388 (16)	−0.0081 (14)	−0.0161 (14)	−0.0124 (14)
C17	0.0480 (16)	0.0388 (15)	0.0277 (13)	−0.0096 (12)	−0.0166 (12)	−0.0036 (11)
O1	0.0574 (12)	0.0413 (11)	0.0281 (10)	−0.0093 (9)	−0.0155 (9)	−0.0099 (8)
O2	0.0470 (11)	0.0442 (11)	0.0349 (10)	−0.0105 (9)	−0.0110 (9)	−0.0173 (9)
O3	0.0514 (12)	0.0668 (14)	0.0488 (12)	0.0087 (10)	−0.0282 (11)	−0.0278 (11)
O4	0.0573 (13)	0.0668 (14)	0.0501 (13)	0.0111 (11)	−0.0320 (11)	−0.0294 (11)
O5	0.0472 (12)	0.0574 (13)	0.0495 (12)	−0.0086 (10)	−0.0220 (10)	−0.0135 (10)
O6	0.088 (3)	0.119 (4)	0.084 (3)	0.033 (3)	−0.061 (3)	−0.060 (3)
O6'	0.083 (9)	0.096 (10)	0.081 (9)	0.016 (7)	−0.052 (7)	−0.032 (7)

### Geometric parameters (Å, º)

**Table:**

Cd1—O1	2.2809 (18)	C6—C7	1.446 (4)
Cd1—O1^i^	2.2809 (18)	C7—H7A	0.9300
Cd1—O2	2.2799 (18)	C8—C9	1.522 (5)
Cd1—O2^i^	2.2799 (18)	C8—H8A	0.9700
Cd1—O4^i^	2.300 (2)	C8—H8B	0.9700
Cd1—O4	2.300 (2)	C9—C10	1.520 (5)
Ni1—O1	2.0098 (19)	C9—H9A	0.9700
Ni1—O2	2.0313 (19)	C9—H9B	0.9700
Ni1—O3	2.080 (2)	C10—H10A	0.9700
Ni1—O5	2.205 (2)	C10—H10B	0.9700
Ni1—N1	2.035 (2)	C11—C12	1.451 (4)
Ni1—N2	2.026 (2)	C11—H11A	0.9300
N1—C7	1.285 (4)	C12—C13	1.403 (4)
N1—C8	1.469 (4)	C12—C17	1.423 (4)
N2—C11	1.271 (4)	C13—C14	1.371 (5)
N2—C10	1.469 (4)	C13—H13A	0.9300
C18—O3	1.228 (4)	C14—C15	1.384 (5)
C18—O4	1.254 (4)	C14—H14A	0.9300
C18—H18	0.9300	C15—C16	1.377 (4)
C1—O1	1.326 (3)	C15—H15A	0.9300
C1—C2	1.394 (4)	C16—C17	1.404 (4)
C1—C6	1.426 (4)	C16—H16A	0.9300
C2—C3	1.391 (4)	C17—O2	1.320 (3)
C2—H2A	0.9300	O5—H5A	0.8500
C3—C4	1.381 (5)	O5—H5B	0.8500
C3—H3A	0.9300	O6—H6A	0.8501
C4—C5	1.365 (5)	O6—H6B	0.8499
C4—H4A	0.9300	O6—H6'B	0.9835
C5—C6	1.401 (4)	O6'—H6'A	0.8500
C5—H5	0.9300	O6'—H6'B	0.8500
			
O2—Cd1—O2^i^	180.0	N1—C7—C6	127.5 (3)
O2—Cd1—O1	73.23 (7)	N1—C7—H7A	116.3
O2^i^—Cd1—O1	106.77 (7)	C6—C7—H7A	116.3
O2—Cd1—O1^i^	106.77 (7)	N1—C8—C9	113.2 (3)
O2^i^—Cd1—O1^i^	73.23 (7)	N1—C8—H8A	108.9
O1—Cd1—O1^i^	180.0	C9—C8—H8A	108.9
O2—Cd1—O4^i^	94.86 (7)	N1—C8—H8B	108.9
O2^i^—Cd1—O4^i^	85.14 (7)	C9—C8—H8B	108.9
O1—Cd1—O4^i^	94.24 (7)	H8A—C8—H8B	107.7
O1^i^—Cd1—O4^i^	85.76 (7)	C10—C9—C8	113.5 (3)
O2—Cd1—O4	85.14 (7)	C10—C9—H9A	108.9
O2^i^—Cd1—O4	94.86 (7)	C8—C9—H9A	108.9
O1—Cd1—O4	85.76 (7)	C10—C9—H9B	108.9
O1^i^—Cd1—O4	94.24 (7)	C8—C9—H9B	108.9
O4^i^—Cd1—O4	180.0	H9A—C9—H9B	107.7
O1—Ni1—N2	173.13 (9)	N2—C10—C9	111.2 (3)
O1—Ni1—O2	84.62 (8)	N2—C10—H10A	109.4
N2—Ni1—O2	88.57 (9)	C9—C10—H10A	109.4
O1—Ni1—N1	90.47 (9)	N2—C10—H10B	109.4
N2—Ni1—N1	96.25 (10)	C9—C10—H10B	109.4
O2—Ni1—N1	173.51 (9)	H10A—C10—H10B	108.0
O1—Ni1—O3	91.60 (8)	N2—C11—C12	127.0 (3)
N2—Ni1—O3	89.79 (9)	N2—C11—H11A	116.5
O2—Ni1—O3	93.73 (8)	C12—C11—H11A	116.5
N1—Ni1—O3	90.65 (9)	C13—C12—C17	119.2 (3)
O1—Ni1—O5	88.29 (8)	C13—C12—C11	117.6 (3)
N2—Ni1—O5	90.61 (9)	C17—C12—C11	123.2 (3)
O2—Ni1—O5	88.68 (8)	C14—C13—C12	122.1 (3)
N1—Ni1—O5	86.92 (9)	C14—C13—H13A	119.0
O3—Ni1—O5	177.57 (8)	C12—C13—H13A	119.0
C7—N1—C8	117.0 (3)	C13—C14—C15	118.7 (3)
C7—N1—Ni1	122.2 (2)	C13—C14—H14A	120.6
C8—N1—Ni1	120.7 (2)	C15—C14—H14A	120.6
C11—N2—C10	118.3 (3)	C16—C15—C14	121.0 (3)
C11—N2—Ni1	123.6 (2)	C16—C15—H15A	119.5
C10—N2—Ni1	118.0 (2)	C14—C15—H15A	119.5
O3—C18—O4	129.4 (3)	C15—C16—C17	121.5 (3)
O3—C18—H18	115.3	C15—C16—H16A	119.2
O4—C18—H18	115.3	C17—C16—H16A	119.2
O1—C1—C2	120.2 (3)	O2—C17—C16	120.9 (3)
O1—C1—C6	121.7 (3)	O2—C17—C12	121.7 (3)
C2—C1—C6	118.1 (3)	C16—C17—C12	117.4 (3)
C3—C2—C1	121.5 (3)	C1—O1—Ni1	124.49 (17)
C3—C2—H2A	119.2	C1—O1—Cd1	134.11 (18)
C1—C2—H2A	119.2	Ni1—O1—Cd1	98.29 (8)
C4—C3—C2	120.2 (3)	C17—O2—Ni1	123.09 (16)
C4—C3—H3A	119.9	C17—O2—Cd1	135.04 (17)
C2—C3—H3A	119.9	Ni1—O2—Cd1	97.69 (7)
C5—C4—C3	119.2 (3)	C18—O3—Ni1	129.3 (2)
C5—C4—H4A	120.4	C18—O4—Cd1	127.57 (19)
C3—C4—H4A	120.4	Ni1—O5—H5A	103.0
C4—C5—C6	122.6 (3)	Ni1—O5—H5B	115.7
C4—C5—H5	118.7	H5A—O5—H5B	103.2
C6—C5—H5	118.7	H6A—O6—H6B	109.5
C5—C6—C1	118.3 (3)	H6A—O6—H6'B	135.0
C5—C6—C7	117.0 (3)	H6B—O6—H6'B	69.8
C1—C6—C7	124.5 (3)	H6'A—O6'—H6'B	109.5

Symmetry code: (i) −*x*, −*y*+1, −*z*+2.

### Hydrogen-bond geometry (Å, º)

**Table:**

*D*—H···*A*	*D*—H	H···*A*	*D*···*A*	*D*—H···*A*
O5—H5*A*···O6′^ii^	0.85	2.04	2.662 (12)	130
O5—H5*B*···O6^ii^	0.85	2.29	2.812 (4)	120
O6—H6*B*···O4^iii^	0.85	1.98	2.737 (4)	147
O6′—H6′*B*···O4^iii^	0.85	2.19	2.769 (12)	125

Symmetry codes: (ii) *x*−1, *y*, *z*; (iii) −*x*+1, −*y*+1, −*z*+2.

## References

[bb1] Bruker (2002). *SADABS*, *SMART* and *SAINT* Bruker AXS Inc., Madison, Winsonsin, USA.

[bb2] Niederhoffer, E. C., Timmons, J. H. & Martell, A. E. (1984). *Chem. Rev.* **84**, 137–203.

[bb3] Sheldrick, G. M. (2008). *Acta Cryst.* A**64**, 112–122. 10.1107/S0108767307043930 18156677

[bb4] Tisato, J., Refosco, F. & Bandoli, F. (1994). *Coord. Chem. Rev.* **135**, 325–397.

[bb5] Wang, X.-Y., Gan, L., Zhang, S.-W. & Gao, S. (2004). *Inorg. Chem.* **43**, 4615–4625. 10.1021/ic0498081 15257590

[bb6] Yamada, S. (1999). *Coord. Chem. Rev.* **192**, 537–557.

[bb7] Zhang, J., Chen, S. M., Valle, H., Wong, M., Austria, C., Cruz, M. & Bu, X. (2007). *J. Am. Chem. Soc.* **129**, 14168–14169. 10.1021/ja076532y PMC5713882 17967029

